# On approximating the modified Bessel function of the second kind

**DOI:** 10.1186/s13660-017-1317-z

**Published:** 2017-02-13

**Authors:** Zhen-Hang Yang, Yu-Ming Chu

**Affiliations:** 1 School of Mathematics and Computation Sciences, Hunan City University, Yiyang, 413000 China; 2 Customer Service Center, State Grid Zhejiang Electric Power Research Institute, Hangzhou, 310009 China

**Keywords:** 33B10, 26A48, modified Bessel function, gamma function, monotonicity

## Abstract

In the article, we prove that the double inequalities

$$ \frac{\sqrt{\pi}e^{-x}}{\sqrt{2(x+a)}}< K_{0}(x)< \frac{\sqrt{\pi }e^{-x}}{\sqrt{2(x+b)}},\qquad 1+ \frac{1}{2(x+a)}< \frac {K_{1}(x)}{K_{0}(x)}< 1+\frac{1}{2(x+b)} $$

hold for all $x>0$ if and only if $a\geq1/4$ and $b=0$ if $a, b\in[0, \infty)$, where $K_{\nu}(x)$ is the modified Bessel function of the second kind. As applications, we provide bounds for $K_{n+1}(x)/K_{n}(x)$ with $n\in\mathbb{N}$ and present the necessary and sufficient condition such that the function $x\mapsto\sqrt {x+p}e^{x}K_{0}(x)$ is strictly increasing (decreasing) on $(0, \infty)$.

## Introduction

The modified Bessel function of the first kind $I_{\nu}(x)$ is a particular solution of the second-order differential equation

$$ x^{2}y^{\prime\prime}(x)+xy^{\prime}(x)- \bigl(x^{2}+ \nu^{2} \bigr)y(x)=0, $$

and it can be expressed by the infinite series

$$ I_{\nu}(x)=\sum_{n=0}^{\infty} \frac{1}{n!\Gamma(\nu+n+1)} \biggl(\frac {x}{2} \biggr)^{2n+\nu}. $$

While the modified Bessel function of the second kind $K_{\nu}(x)$ is defined by

1.1 $$ K_{\nu}(x)=\frac{\pi (I_{-\nu}(x)-I_{\nu}(x) )}{2\sin(\pi\nu)}, $$

where the right-hand side of the identity of (1.1) is the limiting value in case *ν* is an integer.

The following integral representation formula and asymptotic formulas for the modified Bessel function of the second kind $K_{\nu}(x)$ can be found in the literature [1], 9.6.24, 9.6.8, 9.6.9, 9.7.2:

1.2 $$\begin{aligned}& K_{\nu}(x)= \int_{0}^{\infty}e^{-x\cosh(t)}\cosh(\nu t)\,dt\quad (x>0), \end{aligned}$$

1.3 $$\begin{aligned}& K_{0}(x)\sim-\log x\quad (x\rightarrow0), \end{aligned}$$

1.4 $$\begin{aligned}& K_{\nu}(x)\sim\frac{1}{2}\Gamma(\nu) \biggl(\frac{x}{2} \biggr)^{-\nu}\quad (\nu >0 ,x\rightarrow0), \end{aligned}$$

1.5 $$\begin{aligned}& K_{\nu}(x)\sim\sqrt{\frac{\pi}{2x}}e^{-x} \biggl[1+ \frac{4\nu ^{2}-1}{8x}+\frac{ (4\nu^{2}-1 ) (4\nu^{2}-9 )}{2!(8x)^{2}}+\cdots \biggr]\quad (x\rightarrow \infty). \end{aligned}$$

From (1.2) we clearly see that

1.6 $$\begin{aligned}& K_{0}(x)= \int_{0}^{\infty}e^{-x\cosh(t)}\,dt= \int_{1}^{\infty}\frac {e^{-xt}}{\sqrt{t^{2}-1}}\,dt, \end{aligned}$$

1.7 $$\begin{aligned}& K^{\prime}_{0}(x)=- \int_{1}^{\infty}\frac{te^{-xt}}{\sqrt{t^{2}-1}}\,dt=-K_{1}(x). \end{aligned}$$

Recently, the bounds for the modified Bessel function of the second kind $K_{\nu}(x)$ have attracted the attention of many researchers. Luke [2] proved that the double inequality

1.8 $$ \frac{8\sqrt{x}}{8x+1}< \sqrt{\frac{2}{\pi}}e^{x}K_{0}(x)< \frac {16x+7}{(16x+9)\sqrt{x}} $$

holds for all $x>0$.

Gaunt [3] proved that the double inequality

1.9 $$ \frac{1}{\sqrt{x+\frac{1}{2}}}< \frac{\Gamma (x+\frac{1}{2} )}{\Gamma(x+1)}< \sqrt{\frac{2}{\pi}}e^{x}K_{0}(x)< \frac{1}{\sqrt{x}} $$

takes place for all $x>0$, where $\Gamma(x)=\int_{0}^{\infty }t^{x-1}e^{-t}\, dx$ is the classical gamma function.

In [4], Segura proved that the double inequality

1.10 $$ \frac{\nu+\sqrt{x^{2}+\nu^{2}}}{x}< \frac{K_{\nu+1}(x)}{K_{\nu}(x)}< \frac {\nu+\frac{1}{2}+\sqrt{x^{2}+ (\nu+\frac{1}{2} )^{2}}}{x} $$

holds for all $x>0$ and $\nu\geq0$.

Bordelon and Ross [5] and Paris [6] provided the inequality

1.11 $$ \frac{K_{\nu}(x)}{K_{\nu}(y)}>e^{y-x} \biggl(\frac{x}{y} \biggr)^{\nu} $$

for all $\nu>-1/2$ and $y>x>0$.

Laforgia [7] established the inequality

1.12 $$ \frac{K_{\nu}(x)}{K_{\nu}(y)}>e^{y-x} \biggl(\frac{x}{y} \biggr)^{-\nu} $$

for all $y>x>0$ if $\nu\in(0, 1/2)$, and inequality (1.12) is reversed if $\nu\in(1/2, \infty)$.

Baricz [8] presented the inequality

$$ \frac{K_{\nu}(x)}{K_{\nu}(y)}>e^{y-x} \biggl(\frac{x}{y} \biggr)^{-1/2} $$

for all $y>x>0$ and $\nu\in(-\infty, -1/2)\cup(1/2, \infty)$.

Motivated by inequality (1.9), in the article, we prove that the double inequality

$$ \frac{\sqrt{\pi}e^{-x}}{\sqrt{2(x+a)}}< K_{0}(x)< \frac{\sqrt{\pi }e^{-x}}{\sqrt{2(x+b)}} $$

holds for all $x>0$ if and only if $a\geq1/4$ and $b=0$ if $a, b\in [0, \infty)$. As applications, we provide bounds for $K_{n+1}(x)/K_{n}(x)$ with $n\in\mathbb{N}$ and present the necessary and sufficient condition such that the function $x\mapsto\sqrt {x+p}e^{x}K_{0}(x)$ is strictly increasing (decreasing) on $(0, \infty)$.

## Lemmas

In order to prove our main results, we need two lemmas which we present in this section.

### Lemma 2.1

See [9]

*Let* $-\infty\leq a< b\leq\infty$, $f,g:[a,b]\rightarrow{\mathbb{R}}$ *be continuous on* $[a,b]$ *and differentiable on* $(a,b)$, *and* $g'(x)\neq0$ *on* $(a,b)$. *If* $f^{\prime}(x)/g^{\prime}(x)$ *is increasing* (*decreasing*) *on* $(a,b)$, *then so are the functions*

$$ \frac{f(x)-f(a)}{g(x)-g(a)},\qquad \frac{f(x)-f(b)}{g(x)-g(b)}. $$

*If* $f^{\prime}(x)/g^{\prime}(x)$ *is strictly monotone*, *then the monotonicity in the conclusion is also strict*.

### Lemma 2.2

*The function*

2.1 $$ x\mapsto f(x)=\frac{K_{0}(x)}{2 [K_{1}(x)-K_{0}(x) ]}-x $$

*is strictly increasing from* $(0, \infty)$ *onto* $(0, 1/4)$.

### Proof

Let $\omega(t)=\sqrt{(t-1)/(t+1)}$. Then it follows from (1.6), (1.7) and (2.1) that

2.2 $$\begin{aligned}& K_{1}(x)-K_{0}(x)= \int_{1}^{\infty}\omega(t)e^{-xt}\,dt, \\& x\bigl[K_{1}(x)-K_{0}(x)\bigr]=- \int_{1}^{\infty}\omega(t)d \bigl(e^{-xt} \bigr) \\& \hphantom{x\bigl[K_{1}(x)-K_{0}(x)\bigr]}=\omega(t)e^{-xt}|_{t=\infty}^{t=1}+ \int_{1}^{\infty}\omega^{\prime }(t)e^{-xt} \,dt \\& \hphantom{x\bigl[K_{1}(x)-K_{0}(x)\bigr]}= \int_{1}^{\infty}\frac{t-1}{(t^{2}-1)^{3/2}}e^{-xt}\,dt, \\& K_{0}(x)-2x \bigl[K_{1}(x)-K_{0}(x) \bigr]= \int_{1}^{\infty}\frac{\omega (t)}{t+1}e^{-xt}\,dt, \\& f(x)=\frac{K_{0}(x)-2x [K_{1}(x)-K_{0}(x) ]}{2 [K_{1}(x)-K_{0}(x) ]}=\frac{\int_{1}^{\infty} \frac{\omega(t)}{t+1}e^{-xt}\,dt}{2\int_{1}^{\infty}\omega(t)e^{-xt}\,dt}, \\& f^{\prime}(x)=\frac{-\int_{1}^{\infty}\frac{t\omega(t)}{t+1}e^{-xt}\,dt \int_{1}^{\infty}\omega(t)e^{-xt}\,dt+\int_{1}^{\infty}\frac{\omega (t)}{t+1}e^{-xt}\,dt \int_{1}^{\infty}t\omega(t)e^{-xt}\,dt}{2 (\int_{1}^{\infty}\omega (t)e^{-xt}\,dt )^{2}} \\& \hphantom{f^{\prime}(x)}=\frac{\int_{1}^{\infty} (\int_{1}^{\infty}\frac{s-t}{t+1}\omega (t)\omega(s)e^{-x(s+t)}\,dt )\,ds}{ 2 (\int_{1}^{\infty}\omega(t)e^{-xt}\,dt )^{2}} =\frac{\int_{1}^{\infty} (\int_{1}^{\infty}\frac{t-s}{s+1}\omega (s)\omega(t)e^{-x(t+s)}\,ds )\,dt}{ 2 (\int_{1}^{\infty}\omega(t)e^{-xt}\,dt )^{2}} \\& \hphantom{f^{\prime}(x)}=\frac{\int_{1}^{\infty} (\int_{1}^{\infty}\frac{s-t}{t+1}\omega (t)\omega(s)e^{-x(s+t)}\,dt )\,ds +\int_{1}^{\infty} (\int_{1}^{\infty}\frac{t-s}{s+1}\omega(s) \omega(t)e^{-x(t+s)}\,ds )\,dt}{4 (\int_{1}^{\infty}\omega (t)e^{-xt}\,dt )^{2}} \\& \hphantom{f^{\prime}(x)}=\frac{\int_{1}^{\infty}\int_{1}^{\infty}\frac {(s-t)^{2}}{(t+1)(s+1)}\omega(t)\omega(s)e^{-x(t+s)}\,dt\,ds}{ 4 (\int_{1}^{\infty}\omega(t)e^{-xt}\,dt )^{2}}>0 \end{aligned}$$

for all $x>0$.

Note that (1.3)-(1.5) and (2.1) lead to

2.3 $$\begin{aligned}& \lim_{x\rightarrow0}xK_{0}(x)=0,\qquad \lim _{x\rightarrow0}xK_{1}(x)=1, \\& \lim_{x\rightarrow0}f(x)=\lim_{x\rightarrow0} \biggl[ \frac {xK_{0}(x)}{2 (xK_{1}(x)-xK_{0}(x) )}-x \biggr]=0, \end{aligned}$$

2.4 $$\begin{aligned}& \lim_{x\rightarrow\infty}f(x)=\lim_{x\rightarrow\infty} \biggl[ \frac {K_{0}(x)-2x(K_{1}(x)-K_{0}(x))}{2 (K_{1}(x)-K_{0}(x) )} \biggr] =\lim_{x\rightarrow\infty}\frac{\frac{1}{4x}+o (\frac{1}{x} )}{\frac{1}{x}+o (\frac{1}{x} )}= \frac{1}{4}. \end{aligned}$$

Therefore, Lemma 2.2 follows easily from (2.2)-(2.4). □

## Main results

### Theorem 3.1

*Let* $a, b\geq0$. *Then the double inequality*

$$ \frac{1}{\sqrt{x+a}}< \sqrt{\frac{2}{\pi}}e^{x}K_{0}(x)< \frac{1}{\sqrt{x+b}} $$

*holds for all* $x>0$ *if and only if* $a\geq1/4$ *and* $b=0$.

### Proof

Let $x>0$, $f(x)$ be defined by Lemma 2.2, and $f_{1}(x)$, $f_{2}(x)$ and $F(x)$ be respectively defined by

3.1 $$ f_{1}(x)=\frac{\pi}{2}-xe^{2x}K_{0}^{2}(x), \qquad f_{2}(x)=e^{2x}K_{0}^{2}(x) $$

and

3.2 $$ F(x)=\frac{\frac{\pi}{2}-xe^{2x}K_{0}^{2}(x)}{e^{2x}K_{0}^{2}(x)}=\frac {f_{1}(x)}{f_{2}(x)}. $$

Then from (1.5), (1.7) and (3.1) we clearly see that

3.3 $$\begin{aligned}& \lim_{x\rightarrow\infty}f_{1}(x)=\lim_{x\rightarrow\infty}f_{2}(x)=0, \end{aligned}$$

3.4 $$\begin{aligned}& \frac{f^{\prime}_{1}(x)}{f^{\prime}_{2}(x)}=\frac {-e^{2x}K^{2}_{0}(x)+2xe^{2x}K_{0}(x) [K_{1}(x)-K_{0}(x) ]}{ -2e^{2x}K_{0}(x) [K_{1}(x)-K_{0}(x) ]}=f(x). \end{aligned}$$

It follows from (3.2)-(3.4), Lemmas 2.1 and 2.2 together with L’Hôpital’s rule that the function $F(x)$ is strictly increasing on $(0, \infty)$ and

3.5 $$ \lim_{x\rightarrow\infty}F(x)=\frac{1}{4}. $$

Note that (1.3) and (3.2) lead to

3.6 $$ \lim_{x\rightarrow0}F(x)=\lim_{x\rightarrow0} \biggl[\frac{\pi }{2e^{2x}K^{2}_{0}(x)}-x \biggr]=0. $$

Therefore, Theorem 3.1 follows easily from (3.2), (3.5), (3.6) and the monotonicity of $F(x)$. □

### Remark 3.2

From Lemma 2.2 we clearly see that the double inequality

$$ p< \frac{K_{0}(x)}{2 [K_{1}(x)-K_{0}(x) ]}-x< q $$

holds for all $x>0$ if and only if $p\leq0$ and $q\geq1/4$.

From (1.7) and Remark 3.2 we get Corollary 3.3 immediately.

### Corollary 3.3

*Let* $p, q\geq0$. *Then the double inequalities*

$$ 1+\frac{1}{2(x+p)}< \frac{K_{1}(x)}{K_{0}(x)}< 1+\frac{1}{2(x+q)} $$

*and*

3.7 $$ \bigl[\log \bigl(e^{x}\sqrt{x+p} \bigr) \bigr]^{\prime}< -\bigl[\log K_{0}(x)\bigr]^{\prime}< \bigl[ \log \bigl(e^{x}\sqrt{x+q} \bigr) \bigr]^{\prime} $$

*hold for all* $x>0$ *if and only if* $p\geq1/4$ *and* $q=0$.

### Remark 3.4

Let $p\geq0$. Then from inequality (3.7) we know that the function $x\mapsto\sqrt{x+p}e^{x}K_{0}(x)$ is strictly increasing (decreasing) on $(0, \infty)$ if and only if $p=0$ ($p\geq1/4$). We clearly see that the bounds for $K_{1}(x)/K_{0}(x)$ given in Corollary 3.3 are better than the bounds given in (1.10) for $\nu=0$.

From (1.3), (1.5) and Remark 3.4 we get Corollary 3.5 immediately.

### Corollary 3.5

*The double inequality*

3.8 $$ \sqrt{\frac{\pi}{2}}=\lim_{x\rightarrow\infty} \bigl[\sqrt {x+p}e^{x}K_{0}(x) \bigr] < \bigl[\sqrt{x+p}e^{x}K_{0}(x) \bigr]< \lim_{x\rightarrow0} \bigl[\sqrt {x+p}e^{x}K_{0}(x) \bigr]=\infty $$

*holds for all* $x>0$ *if* $p\geq1/4$, *and inequality* (3.8) *is reversed if* $p=0$.

Remark 3.4 also leads to Corollary 3.6.

### Corollary 3.6

*Let* $p, q\geq0$. *Then the double inequality*

$$ \sqrt{\frac{y+p}{x+p}}e^{y-x}< \frac{K_{0}(x)}{K_{0}(y)}< \sqrt{ \frac {y+q}{x+q}}e^{y-x} $$

*holds for all* $0< x< y$ *if and only if* $p\geq1/4$ *and* $q=0$.

### Remark 3.7

We clearly see that the lower bound for $K_{0}(x)/K_{0}(y)$ in Corollary 3.6 is better than the bounds given in (1.11) and (1.12) for $\nu=0$.

### Remark 3.8

From the inequality

$$ \frac{\Gamma (x+\frac{1}{2} )}{\Gamma(x+1)}< \frac{1}{\sqrt {x+\frac{1}{4}}} $$

given in [10], (2.20), and the fact that

$$ \frac{1}{\sqrt{x+\frac{1}{4}}}>\frac{8\sqrt{x}}{8x+1} $$

for all $x>0$ we clearly see that the lower bound given in Theorem 3.1 for $\sqrt{2/\pi}e^{x}K_{0}(x)$ is better than that given in (1.8) and (1.9). But the upper bound given in Theorem 3.1 is weaker than that given in (1.8).

### Remark 3.9

From Theorem 3.1 and Corollary 3.3 we clearly see that there exist $\theta_{1}=\theta_{1}(x)\in(0, 1/4)$ and $\theta_{2}=\theta_{2}(x)\in (0, 1/4)$ such that

$$ K_{0}(x)=\sqrt{\frac{\pi}{2(x+\theta_{1})}}e^{-x},\qquad K_{1}(x)= \biggl[1+\frac{1}{2(x+\theta_{2})} \biggr]\sqrt{ \frac{\pi}{2(x+\theta_{1})}}e^{-x} $$

for all $x>0$.

### Theorem 3.10

*Let* $x>0$, $n\in\mathbb{N}$, $R_{n}(x)=K_{n+1}(x)/K_{n}(x)$, $u_{0}(x)=1+1/(2x)$, $v_{0}(x)=1+1/(2x+1/2)$, *and* $u_{n}(x)$ *and* $v_{n}(x)$ *be defined by*

3.9 $$ u_{n}(x)=\frac{1}{v_{n-1}(x)}+\frac{2n}{x},\qquad v_{n}(x)=\frac {1}{u_{n-1}(x)}+\frac{2n}{x}\quad (n\geq1). $$

*Then the double inequality*

3.10 $$ v_{n}(x)< R_{n}(x)=\frac{K_{n+1}(x)}{K_{n}(x)}< u_{n}(x) $$

*holds for all* $x>0$ *and* $n\in\mathbb{N}$.

### Proof

We use mathematical induction to prove inequality (3.10). From Corollary 3.3 we clearly see that inequality (3.10) holds for all $x>0$ and $n=0$.

Suppose that inequality (3.10) holds for $n=k-1$ ($k\geq1$), that is,

3.11 $$ v_{k-1}(x)< R_{k-1}(x)< u_{k-1}(x). $$

Then it follows from (3.9) and (3.11) together with the formula

$$ \frac{K^{\prime}_{\nu}(x)}{K_{\nu}(x)}=-\frac{K_{\nu-1}(x)}{K_{\nu }(x)}-\frac{\nu}{x}=-\frac{K_{\nu+1}(x)}{K_{\nu}(x)}+ \frac{\nu}{x} $$

given in [11] that

3.12 $$\begin{aligned}& R_{k}(x)=\frac{1}{R_{k-1}(x)}+\frac{2k}{x}, \\& v_{k}(x)=\frac{1}{u_{k-1}(x)}+\frac{2k}{x}< R_{k}(x)< \frac {1}{v_{k-1}(x)}+\frac{2k}{x}=u_{k}(x). \end{aligned}$$

Inequality (3.12) implies that inequality (3.10) holds for $n=k$, and the proof of Theorem 3.10 is completed. □

### Remark 3.11

Let $n=1, 2, 3$. Then Theorem 3.10 leads to

$$\begin{aligned}& \frac{2(x+1)^{2}}{x(2x+1)}< \frac{K_{2}(x)}{K_{1}(x)}< \frac {4x^{2}+9x+6}{x(4x+3)}, \\& \frac{4x^{3}+19x^{2}+36x+24}{x (4x^{2}+9x+6 )}< \frac {K_{3}(x)}{K_{2}(x)}< \frac{2x^{3}+9x^{2}+16x+8}{2x(x+1)^{2}}, \\& \frac{2 (x^{4}+8x^{3}+28x^{2}+48x+24 )}{x (2x^{3}+9x^{2}+16x+8 )}< \frac{K_{4}(x)}{K_{3}(x)} < \frac{4x^{4}+33x^{3}+120x^{2}+216x+144}{x (4x^{3}+19x^{2}+36x+24 )} \end{aligned}$$

for all $x>0$.

### Remark 3.12

It is not difficult to verify that

$$\begin{aligned}& \frac{2(x+1)^{2}}{x(2x+1)}>\frac{1+\sqrt{x^{2}+1}}{x}, \qquad \frac {4x^{2}+9x+6}{x(4x+3)}< \frac{\frac{3}{2}+\sqrt{x^{2}+\frac{9}{4}}}{x}, \\& \frac{4x^{3}+19x^{2}+36x+24}{x (4x^{2}+9x+6 )}>\frac {2+\sqrt{x^{2}+4}}{x},\qquad \frac{2x^{3}+9x^{2}+16x+8}{2x(x+1)^{2}}< \frac{\frac {5}{2}+\sqrt{x^{2}+\frac{25}{4}}}{x}, \\& \frac{2 (x^{4}+8x^{3}+28x^{2}+48x+24 )}{x (2x^{3}+9x^{2}+16x+8 )}>\frac{3+\sqrt{x^{2}+9}}{x}, \\& \frac{4x^{4}+33x^{3}+120x^{2}+216x+144}{x (4x^{3}+19x^{2}+36x+24 )}< \frac{\frac{7}{2}+\sqrt{x^{2}+\frac{49}{4}}}{x} \end{aligned}$$

for $x>0$. Therefore, the bounds given in Remark 3.11 are better than the bounds given in (1.10) for $\nu=1, 2, 3$.
